# A Novel Synthesis of 4-Acetoxyl 5(4*H*)-Oxazolones by Direct α-Oxidation of *N*-Benzoyl Amino-Acid Using Hypervalent Iodine

**DOI:** 10.3390/molecules22071102

**Published:** 2017-07-03

**Authors:** Gang Wen, Wen-Xuan Zhang, Song Wu

**Affiliations:** State Key Laboratory of Bioactive Substance and Function of Natural Medicines, Institute of Materia Medica, Chinese Academy of Medical Sciences and Peking Union Medical College, Beijing 100050, China; wengang@imm.ac.cn

**Keywords:** oxidation, hypervalent iodine, 4-acetoxyl 5(4*H*)-oxazolones, *N*-benzoyl amino-acid, online FTIR

## Abstract

We have developed a new method to prepare 4-acetoxy substituted 5(4*H*)-oxazolones by direct oxidation of *N*-benzoyl amino-acids using hypervalent iodine. The method is efficient, economical and easy to perform for the synthesis of quaternary substituted amino acid derivatives. We used online FTIR monitoring techniques to analyze the reaction, and gave a plausible reaction mechanism.

## 1. Introduction

2-Substituted amino acids and their derivatives are known as structural elements with important physiology [1,2,3]. As an important synthetic intermediate of 2-substituted amino acids, 2-acetoxy-2-amino acids have attracted tremendous interest in organic synthesis [4]. However, to date, chemists have only reported a few methods to synthesize these amino acids, such as oxidating the protected cystinylserine with Pb(OAc)_4_ to the 2-acetoxy compound [5,6,7], using 2-benzamidotrifluorolactic acid as starting materials to yield 4-acetoxy-4-trifluoromethyl-2-phenyloxazol-5(4*H*)-one [8], treating oxazolone with mercuric acetate to form the diacetyl compound [9], transforming 2-acylaminoacrylate with *N*-chlorosuccinimide/HCl/LiOAc in acetic acid to the 2-acetoxy-2-amine acid derivatives [2], converting β-lactams with ruthenium trichloride in the presence of acetaldehyde and acetic acid with molecular oxygen into the corresponding 4-acyloxy β-lactams [10], and using anodic oxidation of 2-ethoxycarbonyl-2-acetamidoacetic acid derivatives, followed by saponification of the one ester groups to 2-alkoxy-2-amino acids and 2-acetoxy-2-amino acids [11]. There are some problems in these methods: 1. It is difficult to prepare the raw materials; 2. The use of metal reagents is expensive and not environmentally-friendly.

Metal-free reagents such as hypervalent iodine compounds have received much attention for low toxicity, mild reaction condition, easy handling and special reactivity compared with heavy metal reagents in organic synthesis reactions. Furthermore, hypervalent iodine reagents have been widely used in various oxidations, such as oxidative dearomatized reactions [12,13], oxidative coupling reactions [14,15], oxidative halogenation reactions [16,17,18], oxidative cyclization reactions [19], oxidative addition [20,21], oxidative amination reaction [22], and so on.

**Scheme 1:**
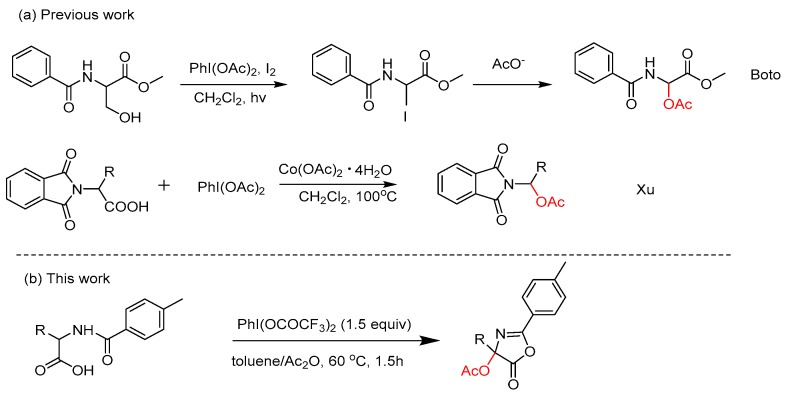
Hypervalent iodine-mediated oxidation of 2-amino acids.

Boto synthesized 2-acetoxy glycine through an unstable α-iodoglycine intermediate, which was then substituted by acetate ions from the reagent PhI(OAc)_2_ (Scheme 1a) [23]. Recently, Xu’s group developed a cobalt-catalyzed decarboxylative C−O bond-forming reaction using hypervalent iodine as oxidizing agent (Scheme 1a). However, these methods involve deiodination or decarboxylative process, which can only give tertiary substituted carbon atoms. Herein, we wish to report a direct oxidation of α-C–H bond of *N*-benzoyl amino-acid using hypervalent iodine (Scheme 1b). With this method, a new synthesis of 4-acetoxy substituted 5(4*H*)-oxazolones was carried out. There are three advantages in our method: 1. Only hypervalent iodine oxidizing agent was used, which was cheap and easy to get; 2. Our method can directly oxidize the α-C–H bond of amino acid derivatives, which does not involve deiodination or decarboxylative process; 3. With this method, quaternary substituted amino acid derivatives could be prepared. 

## 2. Results

Reaction of *N*-benzoyl isoleucine (**1a**) with 1.0 equiv PhI(OCOCF_3_)_2_ in acetic anhydride and toluene (*v*/*v* = 4:1) at 60 °C for 1.5 h gave 4-acetoxy substituted 5(4*H*)-oxazolone (**1b**) as oxidative product in 40% yield (dr = 1:1,Table 1). We found that 1.5 equiv of PhI(OCOCF_3_)_2_ give much better yield, but the increase of PhI(OCOCF_3_)_2_ to 3.0 equivalent afford lower yield (entry 2–3). Replacement of PhI(OCOCF_3_)_2_ with PhI(OAc)_2_ or PhI(OPiv)_2_ led to the reducing of the yield, and the latter gave only 19% yield (entry 5–6). In the absence of PhI(OCOCF_3_)_2_, only oxazolones (**1c**) was afforded, which can give desired product **1b** by the oxidative annulation with addition of PhI(OCOCF_3_)_2_ (see supporting Information). AgF_2_ gave only coupling product without desired product. The temperature also has great influence on this oxidation reaction, which gave lower yields at 30 °C and 90 °C (entry 7–8). Further assessment of the solvent effect indicated that toluene was the best solvent for this reaction, providing higher yield than other commonly used solvents such as ACN, DCE, Acetone and THF (entry 10–13).

With the optional conditions in hand, we then examined the substrate scope of *N-*benzoyl amino acid substrates (Table 2). Both α-alkyl and benzyl substituted *N*-benzoyl amino-acid (**2a**–**10a**) gave desired 4-acetoxyl 5(4*H*)-oxazolones (**2b**–**10b**) in moderated yields with some coupling products in 0–20% yields (except for **1a**, see supporting information). Beside coupling products, α-alkyl substrates **3a** and **5a** also gave rearrangement product *N*-(1-oxo-alkyl)-benzamide in about 20% yields, while substituted *N-*benzoyl amino-acids (**6a**–**10a**) gave some elimination products in about 10–30% yields (see supporting Information). In these conditions, ether and ester groups were very tolerant, as shown in entry 8–9.

## 3. Discussion

**Figure 1:**
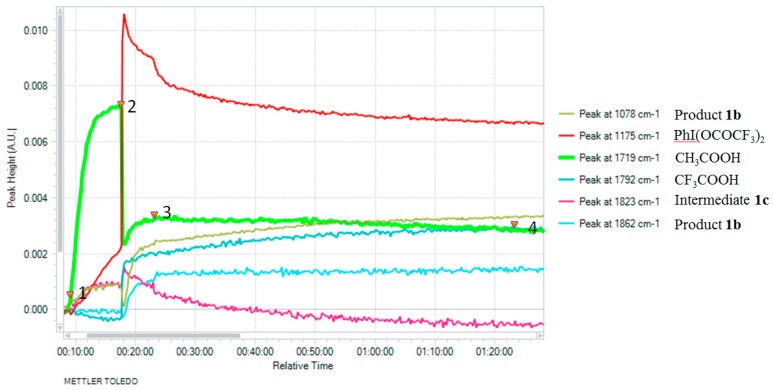
ConcIRT component profiles of oxidative reaction. 1. Added the material; 2. Added the oxidation PhI(OCOCF_3_)_2_; 3, 4. Got sample.

**Figure 2:**
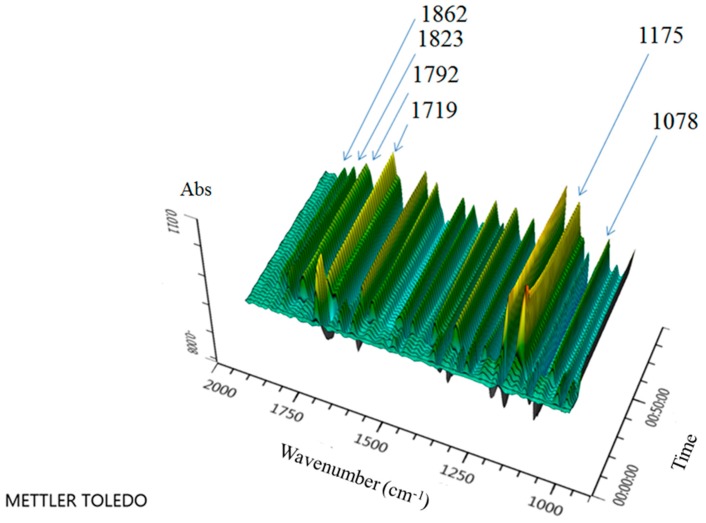
In situ reaction of IR, 3D surface plot of oxidative reaction.

In order to study the reaction kinetics, we used online FTIR techniques (fourier-transform infrared spectra, React IR 15) to monitor the reaction [24,25,26]. At first, we collected the FTIR data of raw material **1a**, the intermediate **1c**, PhI(OCOCF_3_)_2_, acetic acid, trifluoroacetic acid and product **1b** in the solvent (anhydrous toluene:acetic anhydride = 4:1) respectively. To prove the formation of intermediate **1c** before the oxidative annulation, we added the PhI(OCOCF_3_)_2_ in the reaction system 10 min after raw material **1a** and acetic anhydride were heated at 60 °C. In the experiments, ReactIR was continuously used to acquire online IR spectra. The ConcIRT analysis and 3D surface plot tracked changes in absorbance profiles that occur over time of the intermediate **1c**, PhI(OCOCF_3_)_2_, acetic acid, trifluoroacetic acid, and product **1b**, which were shown in Figure 1 and Figure 2.

At first, the peak height at 1822 cm^−1^ (C=O bond, pale red line) increased due to the producing of the intermediate **1c**, and it decreased when we add the oxidant PhI(OCOCF_3_)_2_ to the reaction system. The peak at 1719 cm^−1^ (C=O bond, green line) increased due to the formation of acetic acid, and it reduced because of the ligand exchange with PhI(OCOCF_3_)_2_. The peak at 1792 cm^−1^ (C=O bond, wathet blue line) increased, showing the formation of trifluoroacetic acid. At the same time, the peak at 1078 cm^−1^ (C–O bond, brown line) and 1862 cm^−1^ (C=O bond) began to increase, indicating the formation of the product **1b**. Moreover, the variation trend of ConcIRT showed that the complete reaction needed about 1.5 h (As shown in the Figure 1).

**Scheme 2:**
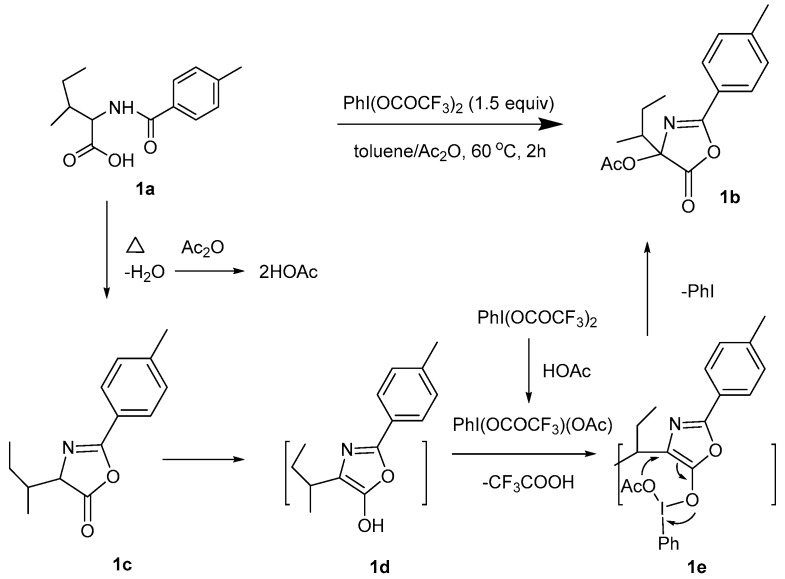
A plausible reaction mechanism of the oxidative annulation reaction.

On the basis of the above experiments, a plausible reaction mechanism was proposed as shown in Scheme 2 [27]. At first, the raw material **1a** was converted to intermediate **1c** [28], which was then transformed to the enoliazted intermediate **1d**. After the ligand exchange of PhI(OCOCF_3_)_2_ with AcOH, the I–O bond of **1e** was formed accompanied by the leave of CF_3_COOH. Then the AcO^−^ added to the double bond accompanied by the elimination of PhI, the final product **1b** was afforded.

## 4. Conclusions

In summary, we have developed a new method to prepare 4-acetoxy substituted 5(4*H*)-oxazolones by direct oxidation of *N*-benzoyl amino-acids using hypervalent iodine. These 4-acetoxy substituted 5(4*H*)-oxazolones can be used as synthetic intermediates for different bioactive compounds or peptidomimetic constituents. The method is efficient, economical and easy to perform for the synthesis of quaternary substituted amino acid derivatives. We used online FTIR monitoring techniques (React IR 15) to monitor the reaction, and gave a plausible reaction mechanism. The synthetic applications of this process are currently underway in our laboratory.

## Figures and Tables

**Table 1:**
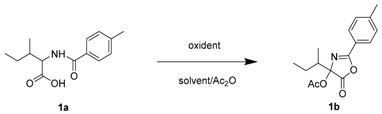
Direct α-oxidation of *N*-benzoyl amino-acid using hypervalent iodine.

Entry	Oxidant (equiv)	Temp (°C)	Solvent	Yield (%) ^a^
1	PhI(OCOCF_3_)_2_ (1)	60	toluene	40
2	PhI(OCOCF_3_)_2_ (1.5)	60	toluene	68 (63 ^b^)
3	PhI(OCOCF_3_)_2_ (3)	60	toluene	50
4	PhI(OCOCF_3_)_2_ (0)	60	toluene	no
5	PhI(OAc)_2_ (1.5)	60	toluene	42
6	PhI(OPiv)_2_ (1.5)	60	toluene	19
7	AgF_2_ (1.5)	60	toluene	no
8	PhI(OCOCF_3_)_2_ (1.5)	30	toluene	38
9	PhI(OCOCF_3_)_2_ (1.5)	90	toluene	43
10	PhI(OCOCF_3_)_2_ (1.5)	60	ACN	50
11	PhI(OCOCF_3_)_2_ (1.5)	60	DCE	43
12	PhI(OCOCF_3_)_2_ (1.5)	60	Acetone	8
13	PhI(OCOCF_3_)_2_ (1.5)	60	THF	no

^a^ Yields were based on 1H NMR analysis of reaction mixture after 1.5 h using (2*E*)-2-Butenedioic acid as standard; ^b^ Isolated yields.

**Table 2:** Substrate scope of *N*-benzoyl amino-acids.

Entry	Substrates	Products (Isolated Yield)	Entry	Substrates	Products (Isolated Yield)
1	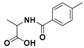 **2a**	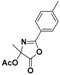 **2b** (53%)	6	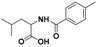 **7a**	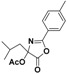 **7b** (47%)
2	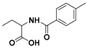 **3a**	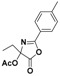 **3b** (47%)	7	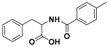 **8a**	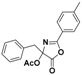 **8b** (45%)
3	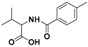 **4a**	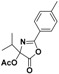 **4b** (53%)	8	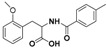 **9a**	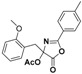 **9b** (51%)
4	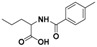 **5a**	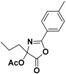 **5b** (47%)	9	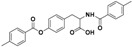 **10a**	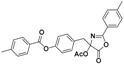 **10b** (45%)
5	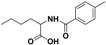 **6a**	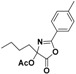 **6b** (60%)			

## Supplementary Materials

Supplementary materials are available online.
