# Supplementary material for: A Novel Synthesis of 4-Acetoxyl 5(4H)-Oxazolones by Direct α-Oxidation of N-Benzoyl Amino-Acid Using Hypervalent Iodine
Source: Molecules. 2017 Jul 3;22(7):1102. doi: 10.3390/molecules22071102 (PMC6152051; doi:10.3390/molecules22071102)
Supplement: Supplementary file 1 [file molecules-22-01102-s001.pdf]

A novel synthesis of 4-acetoxyl 5(*4H*)-oxazolones by direct α-oxidation of N-benzoyl amino-acid using hypervalent iodine

Gang Wen, Wenxuan Zhang,* Song Wu*

State Key Laboratory of Bioactive Substance and Function of Natural Medicines, Institute of Materia Medica, Chinese Academy of Medical Sciences and Peking Union Medical College, Beijing 100050, China

*email: wxzhang@imm.ac.cn; ws@imm.ac.cn

Content

1. Reagent.
2. Instrument.
3. The preparation of N-benzoyl amino acid substrates **1a-10a**.
4. Synthesis of the target compounds **1b-10b**
5. The by-product of the oxidative reaction.
6. The synthesis of the product **1b** from intermediate **1c.**

1. Reagent: Unless otherwise indicated, all solvents and organic reagents were obtained from commercially available sources and were used without further purification. The following solvents were dried using molecular sieves. $PhI(OCOCF_3)_2$ (98%, Innochem), $Pd(OAc)_2$(99%, Innochem), Ac_2O(99+%, Acros) were used in the Pd-catalyzed reaction.

2. Instrument: The reaction process was monitored using thin layer chromatography (TLC) with silica gel plates (thickness = 0.20 mm, GF_{254}) under UV light. Flash chromatography was performed using a ZCX-II, (200-300 mesh) to purify the products. ^{1}H NMR spectra was recorded on a Varian Mercury-500 MHz instrument, while ^{13}C NMR spectra was recorded at 400MHz on a Varian Mercury using DMSO-d_6 as a solvent and tetramethylsilane (TMS) as an internal standard. Mass spectra was obtained using a Waters Acquity UPLC-SQD mass spectrometer. High resolution mass spectra (HRMS) were recorded on an Agilent Technologies LC/MSD TOF spectrometer.

3. The preparation of N-benzoyl amino acid substrates **1a-10a**.

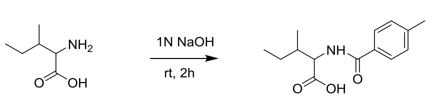

1a-0 **1a**

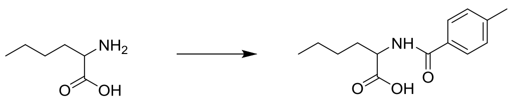

6a-0 **6a**

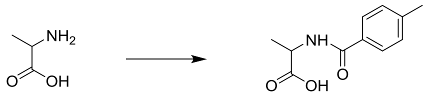

2a-0 **2a**

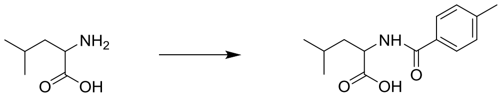

7a-0 **7a**

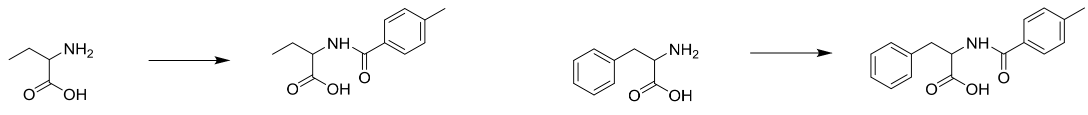

3a-0 3a 8a-0 8a

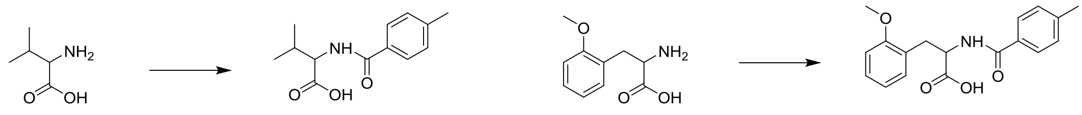

4a-0 4a 9a-0 9a

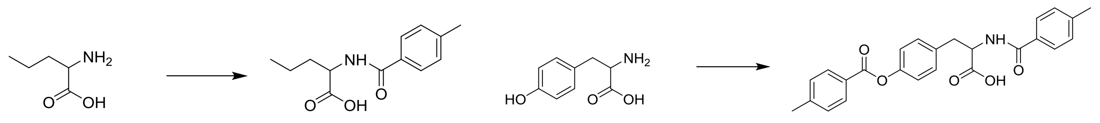

5a-0 5a 10a-0 10a

To a solution of amine (1.0 eq) in 15 ml 1N NaOH at 0 ^{O}C is added 4-Methylbenzoyl chloride dropwise (1.03 eq). The solution is stirred at rt for 2 hours until the reaction is complete based on TLC (PE:EA = 1:1). And then it is quenched with conc. HCl to pH = 2. The precipitate is filtered and the resulting residue is recrystallized in EtOAc/Hex to give the key intermediate as a white solid.

3.1. 3-methyl-2-(4-methylbenzamido)pentanoic acid(**1a**)

White solid; Yield: 92%; ^{1}H NMR (500 MHz, DMSO-d_6) δ: 12.54 (s, 1H), 8.29 (d, *J* = 8.1 Hz, 1H), 7.78 (d, *J* = 7.7 Hz, 2H), 7.26 (d, *J* = 7.8 Hz, 2H), 4.30 (t, *J* = 7.5 Hz, 1H), 2.35 (s, 3H), 1.93 (m, 1H), 1.54 (m, 1H), 1.25 (m, 1H), 0.91 (d, *J* = 6.8 Hz, 3H), 0.85 (t, *J* = 7.5 Hz, 3H); MS (ESI$^+$): m/z 249.40 [M+H]$^+$.

3.2. (4-methylbenzoyl)alanine(**2a**)

White solid; Yield: 85%; ^{1}H NMR (500 MHz, DMSO-d_6) δ: 12.49 (s, 1H), 8.54 (d, *J* = 7.2 Hz, 1H), 7.78 (d, *J* = 7.8 Hz, 2H), 7.26 (d, *J* = 7.8 Hz, 2H), 4.40 (t, *J* = 7.3 Hz, 1H), 2.35 (S, 3H), 1.37 (d, *J* = 7.3 Hz, 3H); MS (ESI$^+$): m/z 207.92 [M+H]$^+$.

3.3. 2-(4-methylbenzamido)butanoic acid(**3a**)

White solid; Yield: 82%; ^{1}H NMR (500 MHz, DMSO-d_6) δ: 12.59 (s, 1H), 8.44 (d, *J* = 7.6 Hz, 1H), 7.79 (d, *J* = 7.7 Hz, 2H), 7.26 (d, *J* = 7.8 Hz, 2H), 4.27 (td, *J* = 8.7, 5.1 Hz, 1H), 2.35 (s, 5H), 1.91 – 1.79 (m, 1H), 1.76 (dt, *J* = 13.6, 7.5 Hz, 1H), 0.93 (t, *J* = 7.3 Hz, 3H); MS (ESI$^+$): m/z 222.38 [M+H]$^+$.

3.4. (4-methylbenzoyl)valine(**4a**)

White solid; Yield: 88%; ^{1}H NMR (500 MHz, DMSO-d_6) δ: 12.59 (s, 1H), 8.28 (d, *J* = 8.2 Hz, 1H), 7.79 (d, *J* = 7.8 Hz, 2H), 7.26 (d, *J* = 7.8 Hz, 2H), 4.26 (t, *J* = 7.6 Hz, 1H), 2.35 (s, 3H), 2.17 (m, 1H), 0.95 (t, *J* = 7.7 Hz, 6H); MS (ESI$^+$): m/z 236.09 [M+H]$^+$.

3.5. 2-(4-methylbenzamido)pentanoic acid(**5a**)

White solid; Yield: 90%; ^{1}H NMR (500 MHz, DMSO-d_6) δ: 12.51 (s, 1H), 8.45 (d, *J* = 7.7 Hz, 1H), 7.78 (d, *J* = 8.0 Hz, 2H), 7.26 (d, *J* = 7.8 Hz, 2H), 4.35 (q, *J* = 7.4 Hz, 1H), 2.35 (s, 3H), 1.75 (q, *J* = 7.6 Hz, 2H), 1.40 (dt, *J* = 14.4, 7.1 Hz, 1H), 1.34 (dd, *J* = 14.0, 7.2 Hz, 1H), 0.88 (t, *J* = 7.4 Hz, 3H); MS (ESI$^+$): m/z 235.96 [M+H]$^+$.

3.6. 2-(4-methylbenzamido)hexanoic acid(**6a**)

White solid; Yield: 87%; ^{1}H NMR (500 MHz, DMSO-d_6) δ: 12.50 (s, 1H), 8.45 (d, *J* = 7.7 Hz, 1H), 7.78 (d, *J* = 7.8 Hz, 2H), 7.26 (d, *J* = 7.8 Hz, 2H), 4.33 (td, *J* = 8.7, 5.3 Hz, 1H), 2.35 (s, 3H), 1.77 (tdd, *J* = 14.2, 12.1, 10.1, 6.8 Hz, 2H), 1.31 (tq, *J* = 15.8, 9.1, 8.5 Hz, 4H), 0.86 (t, *J* = 6.9 Hz, 3H); MS (ESI$^+$): m/z 250.08 $[M+H]^+$.

37. (4-methylbenzoyl)leucine(7**a**)

White solid; Yield: 89%; ^{1}H NMR (400 MHz, DMSO-d_6) δ: 12.67 (s, 1H), 8.41 (d, *J* = 7.9 Hz, 1H), 7.77 (d, *J* = 8.1 Hz, 2H), 7.26 (d, *J* = 7.9 Hz, 2H), 4.47 – 4.28 (m, 1H), 2.34 (s, 3H), 1.73 (m, 2H), 1.56 (m, 1H), 0.90 (d, *J* = 6.3 Hz, 3H), 0.86 (d, *J* = 6.3 Hz, 3H); MS (ESI$^+$): m/z 250.00 $[M+H]^+$.

38. (4-methylbenzoyl)phenylalanine(**8a**)

White solid; Yield: 84%; ^{1}H NMR (500 MHz, DMSO-d_6) δ: 8.40 (d, *J* = 7.2 Hz, 1H), 7.66 (d, *J* = 7.8 Hz, 2H), 7.24 (m, 7.9 Hz, 6H), 7.14 (t, *J* = 7.2 Hz, 1H), 4.50 (s, 1H), 3.17 (dd, *J* = 13.6, 4.0 Hz, 1H), 3.05 (dd, *J* = 13.3, 10.0 Hz, 1H), 2.32 (s, 3H); MS (ESI$^+$): m/z 298.11 $[M+H]^+$.

3.9. 3-(2-methoxyphenyl)-2-(4-methylbenzamido)propanoic acid(**9a**)

White solid; Yield: 85%; ^{1}H NMR (500 MHz, DMSO-d_6) δ: 12.58 (s, 1H), 8.46 (d, *J* = 8.4 Hz, 1H), 7.67 (d, *J* = 8.2 Hz, 2H), 7.27 – 7.19 (m, 3H), 7.16 (t, *J* = 7.8 Hz, 1H), 6.93 (d, *J* = 8.2 Hz, 1H), 6.80 (t, *J* = 7.4 Hz, 1H), 4.62 (m, 1H), 3.79 (s, 3H), 3.24 (dd, *J* = 13.7, 4.6 Hz, 1H), 2.93 (dd, *J* = 13.7, 10.4 Hz, 1H), 2.32 (s, 3H); MS (ESI$^+$): m/z 314.29 $[M+H]^+$.

3.10. 2-(4-methylbenzamido)-3-(4-((4-methylbenzoyl)oxy)phenyl)propanoic acid(**10a**)

White solid; Yield: 50%; ^{1}H NMR (500 MHz, DMSO-d*6*) δ: 8.41 (s, 1H), 7.98 (d, *J* = 7.8 Hz, 2H), 7.68 (d, *J* = 7.8 Hz, 2H), 7.38 (d, *J* = 7.8 Hz, 2H), 7.33 (d, *J* = 8.0 Hz, 2H), 7.24 (d, *J* = 7.9 Hz, 2H), 7.12 (d, *J* = 8.0 Hz, 2H), 4.50 (s, 1H), 3.20 (dd, *J* = 13.6, 9.5 Hz, 1H), 3.09 (dd, *J* = 13.6, 9.5 Hz, 1H), 2.40 (s, 3H), 2.33 (s, 3H); MS (ESI$^+$): m/z 418.32 $[M+H]^+$.

4. Synthesis of the target compounds (**1b**-**10b**)

A mixture of N-benzoylamide amino acid substrates (0.4 mmol, 1.0eq), $PhI(OCOCF_3)_2$ (0.6mmol, 1.5eq) and anhydrous toluene: Acetic anhydride(v: v = 4ml:1ml) in a three-necked flask (10 ml) was heated at 60 ^{o}C for 1.5 hours. After the reaction was completed, the reaction mixture was cooled to rt, and concentrated under reduced pressure. Water was added and the mixture was extracted with DCM. The combined organic layers was washed with brine, dried over anhydrous Na_2SO_4, and concentrated under reduced pressure. The residue was purified by silica gel flash chromatography to give the desired products.

4.1. 4-(sec-butyl)-5-oxo-2-(p-tolyl)-4,5-dihydrooxazol-4-yl acetate(**1b**)

Yellow oil; Yield: 63%; ^{1}H NMR (500 MHz, DMSO-d_6) δ: 7.87 (d, *J* = 7.8 Hz, 2H), 7.40 (d, *J* = 7.8 Hz, 2H), 2.40 (s, 3H), 2.13 (s, 3H), 2.00 (m, 1H), 1.48 (m, 1H), 1.20 (m, 1H), 1.04 (d, *J* = 6.6 Hz, 1.5H), 0.95 – 0.89 (m, 1.5H), 0.86 (m, 3H); ^{13}C NMR (101 MHz, DMSO-d_6) δ: 172.20, 167.96, 164.06, 141.22, 131.66, 128.90, 127.68, 94.46, 42.82, 24.53, 21.24, 21.13, 14.44, 11.26; HRMS Calcd for $C_{16}H_{20}NO_4$ $[M+H^+]$: 290.1392; Found: 290.1380.

4.2. 4-methyl-5-oxo-2-(p-tolyl)-4,5-dihydrooxazol-4-yl acetate(**2b**):

Yellow oil; Yield: 53%; ^{1}H NMR (500 MHz, DMSO-d_6) δ: 7.86 (d, J = 7.5 Hz, 2H), 7.41 (d, *J* = 7.5 Hz, 2H), 2.40 (s, 3H), 2.25 (m, 1H), 2.14 (s, 3H), 1.69 (s, 3H); ^{13}C NMR (101 MHz, DMSO-d_6) δ: 172.29, 168.05, 167.55, 141.31, 131.67, 129.56, 129.37, 128.97, 127.73, 21.29, 21.17; HRMS Calcd for $C_{15}H_{18}NO_4$ [M+H$^+$]: 276.1236; Found: 276.1220.

4.3. 4-ethyl-5-oxo-2-(p-tolyl)-4,5-dihydrooxazol-4-yl acetate(**3b**)

Yellow oil; Yield: 47%; ^{1}H NMR (500 MHz, DMSO-d_6) δ: 7.89 (d, *J* = 7.8 Hz, 2H), 7.42 (d, *J* = 8.0 Hz, 2H), 2.42 (s, 3H), 2.14 (s, 3H), 2.02 (m, 2H), 0.92 (t, *J* = 7.4 Hz, 3H); ^{13}C NMR (101 MHz, DMSO-d_6) δ: 173.57, 170.06, 162.99, 144.82, 130.04, 128.27, 121.83, 92.41, 28.98, 21.48, 20.12, 6.34; HRMS Calcd for $C_{14}H_{16}NO_4$ [M+H$^+$]: 262.1079; Found: 262.1071.

4.4. 4-isopropyl-5-oxo-2-(p-tolyl)-4,5-dihydrooxazol-4-yl acetate(**4b**)

Yellow oil; Yield: 53%; ^{1}H NMR (500 MHz, DMSO-d_6) δ: 7.88 (d, *J* = 7.5 Hz, 2H), 7.41 (d, *J* = 7.5 Hz, 2H), 2.40 (s, 3H), 2.25 (m, 1H), 2.14 (s, 3H), 1.05 (d, *J* = 6.4 Hz, 3H), 0.90 (d, *J* = 6.4 Hz, 3H); ^{13}C NMR (101 MHz, DMSO-d_6) δ: 173.38, 170.13, 163.20, 144.90, 130.08, 128.28, 121.17, 94.29, 34.18, 21.48, 21.40, 15.62. 15.05; HRMS Calcd for $C_{15}H_{18}NO_4$ [M+H$^+$]: 276.1236; Found: 276.1220.

4.5. 5-oxo-4-propyl-2-(p-tolyl)-4,5-dihydrooxazol-4-yl acetate(**5b**)

Yellow oil; Yield: 47%; ^{1}H NMR (500 MHz, DMSO-d_6) δ: 7.87 (d, *J* = 7.9 Hz, 2H), 7.40 (d, *J* = 7.9 Hz, 2H), 2.40 (s, 3H), 2.12 (s, 3H), 2.00 (m, 1H), 1.91 (m, 1H), 1.35 (m, 2H), 0.90 (t, *J* = 7.4 Hz, 3H); ^{13}C NMR (101 MHz, DMSO-d_6) δ: 173.68, 170.02, 162.92, 144.81, 130.03, 128.26, 121.86, 92.01, 37.51, 21.47, 20.13, 15.28, 13.74; HRMS Calcd for $C_{15}H_{18}NO_4$ [M+H$^+$]: 276.1236; Found: 276.1226.

4.6. 4-butyl-5-oxo-2-(p-tolyl)-4,5-dihydrooxazol-4-yl acetate(**6b**)

Yellow oil; Yield: 60%; ^{1}H NMR (500 MHz, DMSO-d_6) δ: 7.87 (d, *J* = 8.1 Hz, 2H), 7.40 (d, *J* = 7.9 Hz, 2H), 2.40 (s, 3H), 2.12 (s, 3H), 2.00 (m, 1H), 1.96 (m, 1H), 1.32 (m, 4H), 0.84 (t, *J* = 6.6 Hz, 3H); ^{13}C NMR (126 MHz, DMSO-d_6) δ: 173.63, 169.98, 162.89, 144.80, 130.02, 128.25, 121.84, 92.02, 35.25, 23.74, 21.93, 21.44, 20.10, 13.80; HRMS Calcd for $C_{16}H_{20}NO_4$ [M+H$^+$]: 290.1392; Found: 290.1378.

4.7. 4-isobutyl-5-oxo-2-(p-tolyl)-4,5-dihydrooxazol-4-yl acetate(**7b**)

Yellow oil; Yield: 47%; ^{1}H NMR (500 MHz, DMSO-d_6) δ: 7.88 (d, *J* = 8.1 Hz, 2H), 7.42 (d, *J* = 7.9 Hz, 2H), 2.42 (s, 3H), 2.13 (s, 3H), 2.12 (s, 1H), 1.97 (m, 1H), 1.90 (m, 1H), 1.83 (m, 1H), 0.96 (t, *J* = 6.0 Hz, 6H); ^{13}C NMR (101 MHz, DMSO-d_6) δ: 172.20, 167.94, 163.31, 141.21, 131.67, 128.90, 127.68, 92.02, 47.17, 23.75, 22.38, 21.24, 21.12, 14.14; HRMS Calcd for $C_{16}H_{20}NO_4$ [M+H$^+$]: 290.1392; Found: 290.1390.

4.8. 4-benzyl-5-oxo-2-(p-tolyl)-4,5-dihydrooxazol-4-yl acetate(**8b**)

Yellow oil; Yield: 45%; ^{1}H NMR (500 MHz, DMSO-d_6) δ: 7.74 (d, *J* = 7.9 Hz, 2H), 7.35 (d, *J* = 7.9 Hz, 2H), 7.20 (q, *J* = 7.3 Hz, 5H), 3.44 (d, *J* = 13.3 Hz, 1H), 3.31 (d, *J* = 13.3 Hz, 1H), 2.37 (s, 3H), 2.15 (s, 3H); ^{13}C NMR (101 MHz, DMSO-d_6) δ: 172.21, 167.96, 166.52, 142.05, 141.23, 135.14, 131.66, 129.44, 128.91, 128.50, 127.68, 109.72, 30.19, 21.25, 21.13; HRMS Calcd for $C_{19}H_{18}NO_4$ [M+H$^+$]: 324.1236; Found: 324.1234.

4.9. 4-(2-methoxybenzyl)-5-oxo-2-(p-tolyl)-4,5-dihydrooxazol-4-yl acetate(**9b**)

Yellow oil; Yield: 51%; ^{1}H NMR (500 MHz, DMSO-d_6) δ: 7.72 (d, *J* = 7.9 Hz, 2H), 7.34 (d, *J* = 7.9 Hz, 2H), 7.20

– 7.12 (m, 2H), 6.89 (d, *J* = 8.2 Hz, 1H), 6.80 (t, *J* = 7.4 Hz, 1H), 3.69 (s, 3H), 3.44 – 3.32 (q, 2H), 2.36 (s, 3H), 2.13 (s, 3H); ^{13}C NMR (101 MHz, DMSO-d_6) δ: 172.85, 169.88, 162.39, 157.94, 144.64, 132.21, 131.70, 129.97, 129.51, 129.36, 128.90, 128.85, 127.98, 127.68, 121.81, 120.13, 119.75, 111.16, 92.43, 55.54, 35.34, 21.44, 20.21; HRMS Calcd for $C_{20}H_{20}NO_5$ [M+H$^+$]: 354.1341; Found: 354.1322.

4.10. 4-((4-acetoxy-5-oxo-2-(p-tolyl)-4,5-dihydrooxazol-4-yl)methyl)phenyl 4-methylbenzoate(**10b**)
Yellow oil; Yield: 45%; ^{1}H NMR (500 MHz, DMSO-d_6) δ: 7.97 (d, *J* = 7.8 Hz, 2H), 7.79 (d, *J* = 7.9 Hz, 2H), 7.42 – 7.34 (m, 4H), 7.29 (d, *J* = 8.1 Hz, 2H), 7.15 (d, *J* = 8.1 Hz, 2H), 3.46 (d, *J* = 13.4 Hz, 1H), 3.36 (d, *J* = 13.4 Hz, 1H), 2.40 (s, 3H), 2.38 (s, 3H), 2.16 (s, 3H); ^{13}C NMR (101 MHz, DMSO-d_6) δ: 173.01, 169.93, 164.59, 162.96, 150.22, 144.88, 144.71, 132.11, 130.05, 130.00, 129.69, 129.50, 129.46, 128.18, 127.67, 126.26, 121.79, 92.29, 40.59, 21.48, 21.42, 20.17; HRMS Calcd for $C_{27}H_{24}NO_6$ [M+H$^+$]: 458.1604; Found: 458.1587.

5. The by-product of the oxidative reaction.

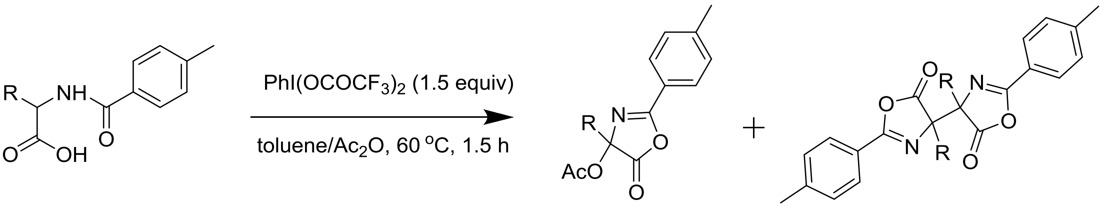

I II Ⅲ

Entry	Substrates	Other by-product	Ⅲ(Coupling)
1	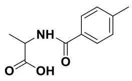 2a		16%[a]
2	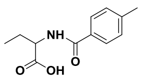 3a	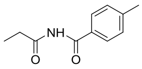 (25%)	16%
3	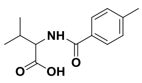 4a		40%
4	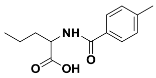 5a	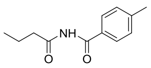 (22%)	20%
5	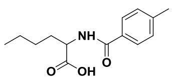 6a		20 %
6	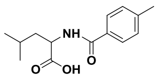 7a	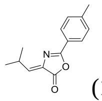 (10%)	40%
7	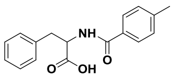 8a		10%

		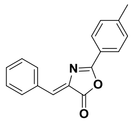 (28%)	
8	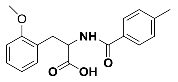 9a	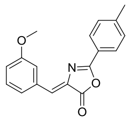 (30%)b	18%
9	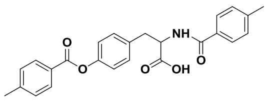 10a	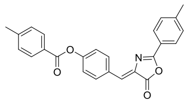 (30%)[b]	

[a] Isolated yields

[b] Yields were based on UPLC-MS

6. The synthesis of the product **1b** from intermediate **1c.**

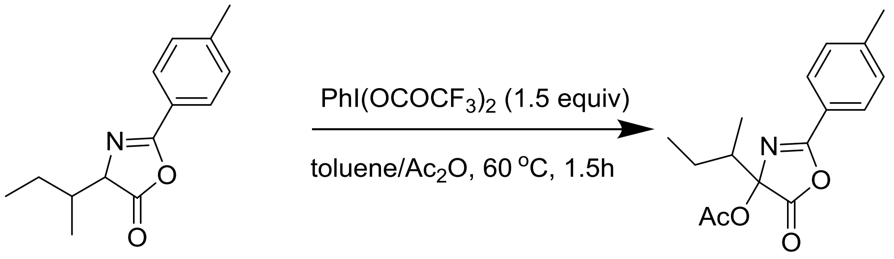

60%

1c **1b**

A mixture of 4-(sec-butyl)-2-(p-tolyl)oxazol-5(*4H*)-one **1c** (0.4 mmol, 1.0eq), $PhI(OCOCF_3)_2$ (0.6mmol, 1.5eq) and anhydrous toluene: Acetic anhydride(v: v = 4ml:1ml) in a three-necked flask (10 ml) was heated at 60 °C for 1.5 hours. After the reaction was completed, the reaction mixture was cooled to rt, and concentrated under reduced pressure. Water was added and the mixture was extracted with DCM. The combined organic layers was washed with brine, dried over anhydrous Na_2SO_4, and concentrated under reduced pressure. The residue was purified by silica gel flash chromatography to give the desired products **1b**.
